# Role of anoikis-related gene PLK1 in kidney renal papillary cell carcinoma: a bioinformatics analysis and preliminary verification on promoting proliferation and migration

**DOI:** 10.3389/fphar.2023.1211675

**Published:** 2023-06-29

**Authors:** Li Gan, Qiyu Xiao, Yusong Zhou, Ying Fu, Mengjie Tang

**Affiliations:** ^1^ Department of Anesthesiology, Hunan Cancer Hospital, The Affiliated Cancer Hospital of Xiangya School of Medicine, Central South University, Changsha, China; ^2^ Department of Nuclear Medicine, Hunan Cancer Hospital, The Affiliated Cancer Hospital of Xiangya School of Medicine, Central South University, Changsha, China; ^3^ Department of Pharmacy, The Third Xiangya Hospital, Central South University, Changsha, China; ^4^ Department of Pathology, Hunan Cancer Hospital, The Affiliated Cancer Hospital of Xiangya School of Medicine, Central South University, Changsha, China

**Keywords:** anoikis, KIRP, PLK1, immune microenvironment, proliferation, migration

## Abstract

**Background:** Kidney renal papillary cell carcinoma (KIRP) is a rare malignancy with a very poor prognosis. Anoikis is a specific form of apoptosis involved in carcinogenesis, but the role of anoikis in KIRP has not been explored.

**Methods:** Anoikis-related genes (ARGs) were obtained from the GeneCards database and Harmonizome database and were used to identify different subtypes of KIRP and construct a prognostic model of KIRP. In addition, we also explored the immune microenvironment and enrichment pathways among different subtypes by consensus clustering into different subtypes. Drug sensitivity analysis was used to screen for potential drugs. Finally, we verified the mRNA and protein expression of the independent prognostic gene PLK1 in patient tissues and various cells and further verified the changes in relevant prognostic functions after constructing a PLK1 stable knockdown model using ShRNA.

**Results:** We identified 99 differentially expressed anoikis-related genes (DEGs) associated with KIRP survival, and selected 3 genes from them to construct a prognostic model, which can well predict the prognosis of KIRP patients. Consensus clustering divided KIRP into two subtypes, and there was a significant difference in survival rates between the two subtypes. Immune profiling revealed differing immune statuses between the two subtypes, and functional analysis reveals the differential activity of different functions in different subtypes. Drug sensitivity analysis screened out 15 highly sensitive drugs in the high-risk group and 11 highly sensitive drugs in the low-risk group. Univariate and multivariate Cox regression analysis confirmed that PLK1 was an independent prognostic factor in KIRP, and its mRNA and protein expression levels were consistent with gene differential expression levels, both of which were highly expressed in KIRP. Functional verification of PLK1 in KIRP revealed significant results. Specifically, silencing PLK1 inhibited cell proliferation, clonogenicity, and migration, which indicated that PLK1 plays an important role in the proliferation and migration of KIRP.

**Conclusion:** The prognosis model constructed by ARGs in this study can accurately predict the prognosis of KIRP patients. ARGs, especially PLK1, play an important role in the development of KIRP. This research can help doctors provide individualized treatment plans for KIRP patients and provide researchers with new research ideas.

## 1 Introduction

Kidney renal papillary cell carcinoma (KIRP) is aggressive carcinoma with a low incidence and poor prognosis. KIRP accounts for approximately 10%–20% of renal parenchymal tumors (Fernandes and Lopes, 2015) and is the most common subtype of non-clear cell renal cell carcinoma (Hu et al., 2019). According to a study conducted between 1992 and 2015 using the Surveillance, Epidemiology, and End Results (SEER) database, the incidence of KIRP is approximately 0.934 per 100,000 patients (Saad et al., 2019). KIRP mainly occurs in male and black patients, and its prognosis is poor. Notably, type 2 KIRP may have a worse prognosis than type 1 KIRP (Sweeney et al., 2022). However, past studies have found no successful cases of targeted therapy against KIRP (Akhtar et al., 2019). Thus, the search for potential therapeutic targets and prognostic markers for KIRP has become a key step in its treatment.

Anoikis is a type of programmed cell death, apoptosis induced by loss of or inappropriate cell adhesion (Valentijn et al., 2004). Anoikis results from insufficient cell-matrix interactions, which disrupt the multiple cytokine networks for growth, motility, and angiogenesis that the extracellular matrix provides to cells through enzymatic digestion and cytoskeletal remodeling (Frisch and Ruoslahti, 1997; Cao et al., 2016). Anoikis is involved in a variety of pathological processes, including carcinogenesis. Tumor cells are able to promote anoikis resistance primarily by regulating integrins and initiating epithelial-mesenchymal transition (EMT). Tumor cells metastasize or migrate to distant sites following EMT or the extracellular matrix (ECM) dissection processes, colonize and proliferate at new sites, and ultimately lead to tumor spread and a loss of surgical intervention opportunities (Guan, 2015). Tumor cells can employ multiple mechanisms to eliminate anoikis and facilitate their invasion and metastasis. Tumor cells can promote anoikis resistance by activating oncogenic signaling of pro-survival pathways, or through changes in the acidic environment and generation of reactive oxygen species (ROS) within the tumor microenvironment (Hu et al., 2019; Vander Linden and Corbet, 2019; Wang et al., 2019). While anoikis has been widely studied in many tumors, including colorectal carcinoma (Cai and Zhou, 2022), head and neck squamous cell carcinoma (Chi et al., 2022), and clear cell renal cell carcinoma (Chen et al., 2022), its role in KIRP remains unknown.

In this study, we continued to explore the TCGA database and GEO database to study the prognosis and immune correlation of anoikis-related genes (ARGs) in KIRP and screened out the independent prognostic gene PLK1 for bioinformatics analysis and preliminary verification of its role in promoting proliferation and migration, thus further clarifying the role of ARGs in KIRP. This study can provide new insights and perspectives for the treatment strategies and anti-tumor targets of KIRP patients.

## 2 Methods and materials

### 2.1 Data download and its preprocessing

On 20 December 2022, we downloaded 290 KIRP samples from The Cancer Genome Atlas (TCGA) database; Supplementary Table S1 presents relevant clinical information. From The Cancer Genome Atlas-Kidney Renal Papillary Cell Carcinoma Database (TCGA-KIRP, https://portal.gdc.cancer.gov/), we downloaded 323 samples and clinical data from 291 KIRP patients. RNA-Seq expression data must be normalized using the FPKM (Fragments Per Kilobase of exon model per Million mapped fragments) method. From the Gene Expression Omnibus (GEO, https://www.ncbi.nlm.nih.gov/gds) database, we obtained the GSE2748 dataset, which includes clinical information from 34 tumor specimens. Perl scripts and R (version 4.2.2) with the R Bioconductor packages were used to perform the data analysis.

### 2.2 Identification and mutation frequency analysis of ARGs

ARGs were obtained from the GeneCards database (https://www.genecards.org/) with a correlation score >0.4 as a filter and the Harmonizome database (https://maayanlab.cloud/Harmonizome/) (Rouillard et al., 2016) (Supplementary Table S2). Based on these ARGs, we used the “limma” package to select differentially expressed genes (DEGs) between normal and tumor samples in the training dataset (|log FC| > 1, *p*-value <0.05). Subsequently, the search tool for the retrieval of interacting genes (STRING, https://string-db.org) was used to construct protein-protein interaction (PPI) networks for 99 ARGs to retrieve interacting genes. We then selected prognosis-related genes using univariate Cox analysis. Lasso analysis was applied to remove overfitting genes. The positions of copy number variations (CNV) alterations in 11 ARGs on 23 chromosomes were mapped by using the “RCircos” package in R.

### 2.3 Consensus cluster analysis

With the R package “ConsensusClusterPlus”, 11 prognostic ARGs were used to perform consensus clustering analysis with a maximum of K = 9. Clustering is performed based on partitioning around the center point using ‘Euclidean’ distance and is validated 1000 times. Next, KIRP patients were divided into different molecular subtypes for further analysis according to the best classification with K = 2-9.

### 2.4 Constructing a prognostic model

The R package “glmnet” was used to select ARGs associated with overall survival (OS) based on univariate Cox regression analysis and utilizing the Least Absolute Shrinkage and Selection Operator (LASSO) algorithm. Next, multivariate Cox regression analysis was performed to select ARGs that could independently predict the prognosis of KIRP. A risk model was established based on the prognostic features of the ARGs using the following formula: prognostic risk score = $\sum_{i=1}^{n}\exp-genei*coef-genei$ . KIRP patients were subsequently divided into low-risk and high-risk groups based on the median risk score. By using the R package “survival”, Kaplan-Meier survival curves were used to estimate the OS rate of KIRP patients in the low-risk and high-risk groups.

### 2.5 Constructing a nomogram

Then, combining the risk score and other clinicopathological data, we used the R packages “rms” and “regplot” to construct a nomogram. In the nomogram scoring system, each variable is assigned a score. The total score for each sample is calculated by summing the scores of all variables, enabling the prediction of the 1-y, 3-y, and 5-year survival rates of KIRP patients. Calibration nomograms were used to depict the correlation between predicted 1-y, 3-y, and 5-year survival events and actual observed outcomes. The R package “pROC” was used to evaluate the diagnostic accuracy of the risk score and clinicopathological features of KIRP.

### 2.6 Immune microenvironmental landscape and drug sensitivity analysis

ESTIMATE (Estimation of Stromal and Immune cells in Malignant Tumours using Expression data) was used to assess the immune cell abundance (ImmuneScores) and stromal cell abundance (StromalScores). We estimated KIRP’s stromal, immune, and ESTIMATE scores by using the R package “estimate”. Based on “CIBERSORT R script v1.03″, the proportions of 22 types of immune cells were estimated using the CIBERSORT algorithm. The R package “GSVA” was used to perform single-sample gene set enrichment analysis (ssGSEA) to evaluate the differences in expression of 23 immune cells. The immune function score of each patient was evaluated by the R package “GSVA”. The Genomics of Drug Sensitivity in Cancer (GDSC) database was used to predict antitumor drug responses in the low-risk and high-risk groups for each KIRP sample, with drug sensitivity (IC50) serving as an important indicator for evaluating treatment response. The R package “ggplot2” was used to visualize all statistical analyzes.

### 2.7 Screening for independent prognostic genes

Univariate and multivariate Cox regression analysis and using forest plot by “forestplot” package to display each variable (*p*-value, hazard ratios (HR), and 95% confidence intervals (CI)), screening independent prognostic genes. The expression levels of independent prognostic genes in KIRP tissues and adjacent normal tissues were compared using the R package “ggplot2”. Kaplan-Meier survival curves yielded *p*-values and HR with 95% CI by log-rank test and univariate Cox regression for comparison between high and low expression of independent prognostic genes. *P* < 0.05 was considered statistically significant.

### 2.8 Tissue samples

During January and March 2023, 10 pairs of KIRP tissues and adjacent normal tissues were collected from the Third Xiangya Hospital of Central South University. These tissues were used to detect PLK1 expression levels by immunohistochemistry. The study has been approved by the Ethics Committee of the Third Xiangya Hospital of Central South University. The approval number is 23188.

### 2.9 Immunohistochemical staining

Adjacent normal tissues and KIRP tissues were fixed in 4% paraformaldehyde and then embedded in paraffin and sectioned to a thickness of 6 μm. For immunohistochemical staining, sections were deparaffinized and rehydrated. Rehydrated sections were treated with Tris-EDTA buffer solution containing 10 mM Tris-Hcl and 1 mM EDTA to expose antigens and inactivate endogenous peroxidases. The sections were then boiled in a pressure cooker for 5 min. After being washed 3 times, sections were incubated with BSA for 30 min and subsequently incubated with anti-PLK1 antibody (1:200 dilution; proteintech, China) overnight at 4°C. The following day, sections were incubated at room temperature with a secondary antibody for 1 h, then developed with DAB staining solution for 30 s. After the nucleus was stained with hematoxylin, sections were dehydrated and mounted using neutral resins.

### 2.10 Quantitative real-time PCR

Total RNA was isolated using an RNeasy mini kit (QIAGEN, Beijing, China). High-capacity cDNA reverse transcription kit (Thermo, Shanghai, China) was used to synthesize the complementary DNA (cDNA). PCR was carried out using TaqMan Gene Expression Master Mix (Bio-Rad, Shanghai, China) according to the manufacturer’s protocol and TaqMan probes for human PLK1 and GAPDH (Sangon Biotech, Shanghai, China). Primers used in this study included PLK1 (forward 5′-GTG​CCT​AAG​TCT​CTG​CTG​CTC​AAG-3′, reverse 5′-TCC​AAC​ACC​ACG​AAC​ACG​AAG​TC-3′), GAPDH (5′-CAG​GAG​GCA​TTG​CTG​AT-3′, 5′-GAA​GGC​TGG​GGC​TCA​TTT-3′).

### 2.11 Western blot

The cells were transfected with PLK1 or control plasmid for 48h, and the protein was extracted. The protein extract was separated by SDS-PAGE and transferred to a PVDF membrane, which was then probed with an anti-PLK1 antibody (1:1000 dilution; proteintech, China). The antigen-antibody response was observed by a ChemiDoc system using a peroxide-coupled secondary antibody. ImageJ was used to quantify the intensity of the band.

### 2.12 MTT

Cells were transfected with PLK1 or a control plasmid for 24 h and then seeded into a 96-well plate at a density of 6 × 10^3 cells per well. 50 μM of 2 mg/mL MTT was added to each well and incubated in the incubator for 4 h. After removing the medium, 150 μL DMSO was added and shaken for 10 min. Absorbance (OD) was measured at 490 nm.

### 2.13 Scratch assay

Cells were transfected with PLK1 or a control plasmid for 24 h and then seeded into a 12-well plate at a density of 3 × 10^5 cells per well. After 24 h, cells were scraped using a 200 µL tip to create an injured area. Cells were then washed twice with phosphate-buffered saline and incubated with serum-free medium for the indicated time periods. Digital images were taken under the microscope at times 0 and 24 h.

### 2.14 Transwell assay

After 24 h of transfection with PLK1 or a control plasmid, 4 × 10^4 cells in 200 μL of serum-free medium were seeded into the upper chamber of a Transwell device. 600 μL medium supplemented with 10% serum was used as a chemoattractant in the lower chamber. After 24 h, cells were fixed with 4% paraformaldehyde and stained with 0.1% crystal violet. Uninvaded cells on the upper sides of the membrane were removed with absorbent cotton and then photographed under a microscope.

## 3 Results

### 3.1 Identification of DEGs in KIRP and adjacent normal tissues

**FIGURE 1:**
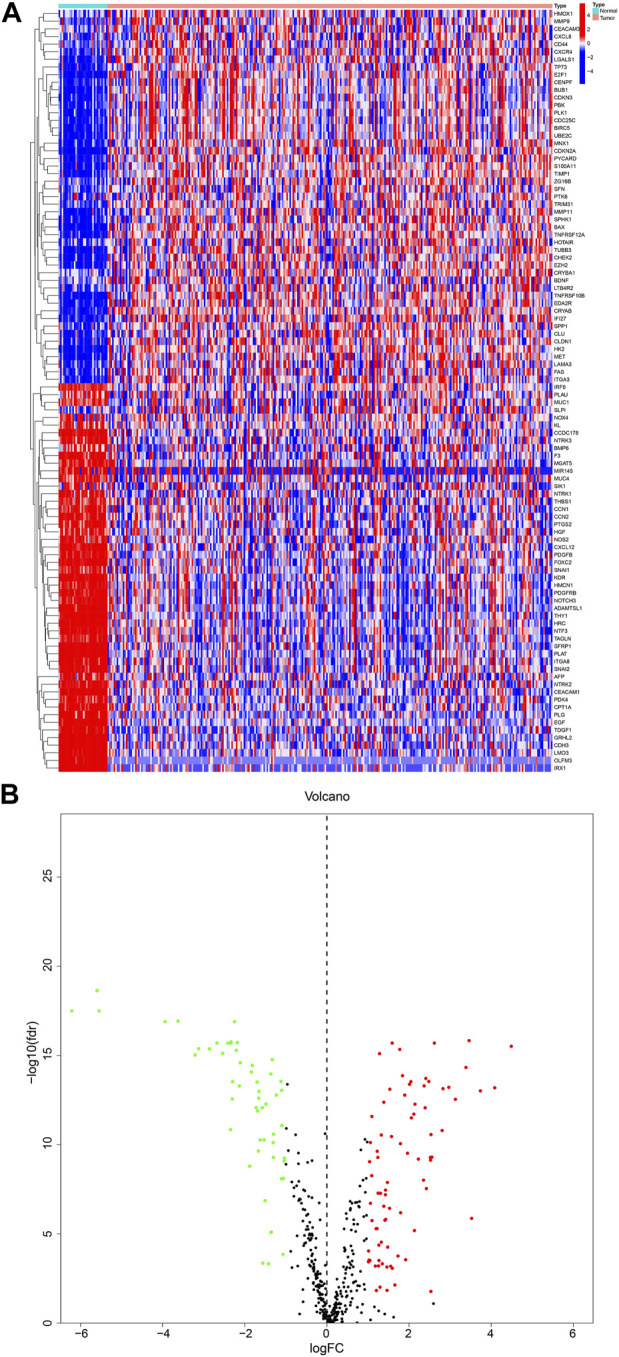
DEGs in KIRP and adjacent normal tissues **(A)** Heatmap showing 99 DEGs in KIRP. **(B)** Volcano diagram shows the DEGs with the threshold set at |FC| ≥ 2 and *p*-value <0.05.

We obtained a total of 640 ARGs from the Genecards database and the Harmonizome database. From the TCGA database, we obtained a total of 135 genes expressed in KIRP and adjacent normal tissues. Visualization of these genes resulted in the identification of 99 DEGs, with 50 genes upregulated and 49 downregulated in KIRP, as shown in the heatmap and volcano diagram (Figures 1A, B). Finally, a protein-protein interaction (PPI) analysis was used to reveal the key nodes of 99 DEGs. We found that PLK1, CDKN3, PDK4, CDKN1A, TAGLN, etc. were important central genes (Supplementary Figure S1).

### 3.2 Screening of prognostic-related genes and mutation frequency analysis

**FIGURE 2:**
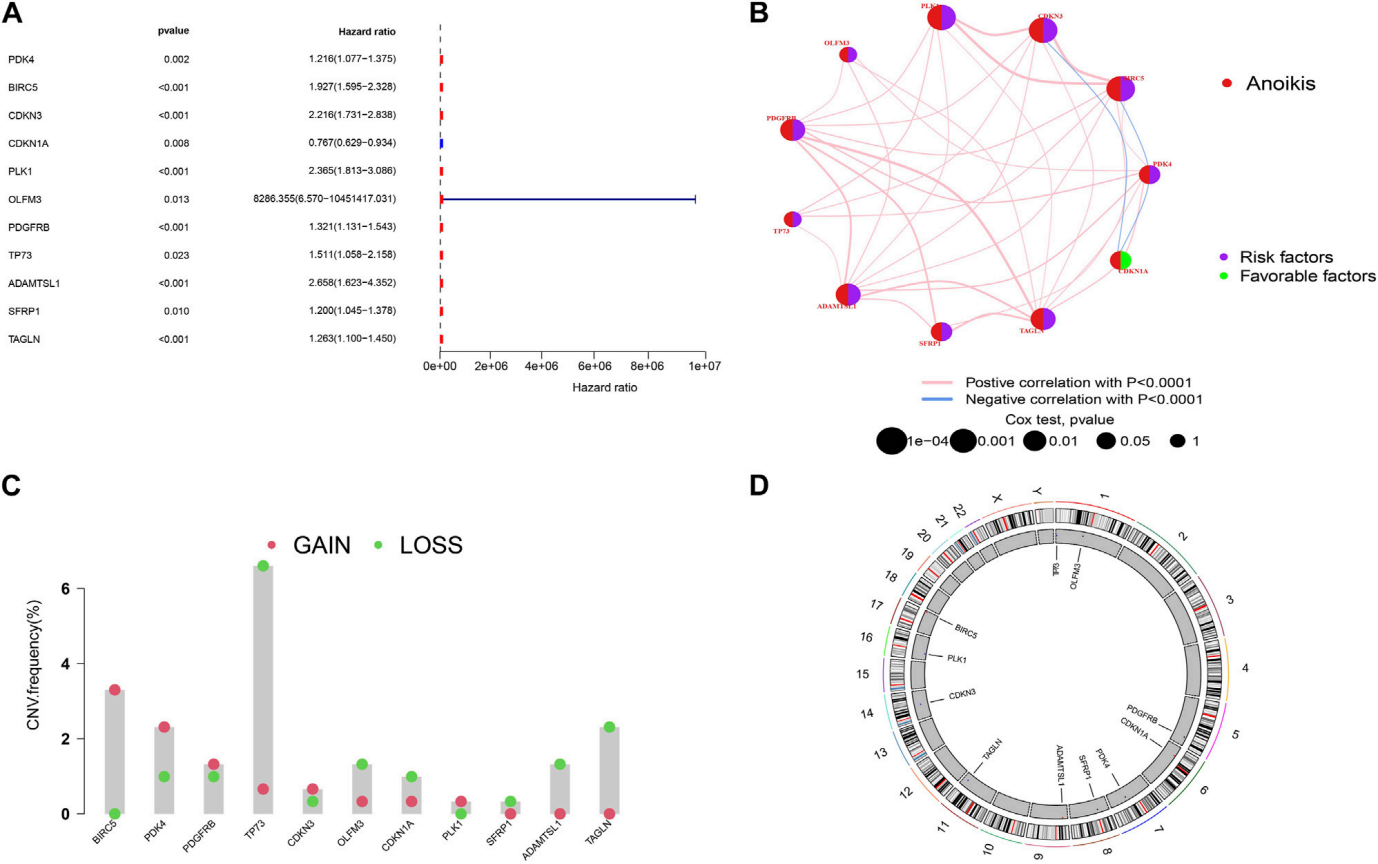
Screening of prognostic-related genes and mutation frequency analysis **(A)**The forest plot shows the top 11 ARGs (*p* < 0.01) via the univariate Cox regression analysis. **(B)** Network diagram showed the correlations between the top 11 ARGs. **(C)** Copy number variations (CNVs) of 11 ARGs in TCGA-KIRP. **(D)** Chromosome region and alteration of ARGs.

To establish a more accurate signature of ARGs, we used univariate regression analysis on the 99 DEGs, identifying 11 genes (PDK4, BIRC5, CDKN3, CDKN1A, PLK1, OLFM3, PDGFRB, TP73, ADAMTSL1, SFRP1, TAGLN) associated with KIRP prognosis (Figure 2A). The network diagram shows that 10 of these 11 genes are high-risk genes, while CDKN1A is a low-risk gene, and there are positive regulatory relationships among most genes, while there are negative regulatory relationships between CDKN3 and CDKN1A, CDKN1A and PDK4, and PDK4 and BIRC5 (Figure 2B). Figure 2C shows that the frequency of copy number gain of BIRC5, PDK4, PDGFRB, CDKN3, and PLK1 is greater than that of copy number loss, while the frequency of copy number gain of TP73, OLFM3, CDKN1A, SFRP1, ADAMTSL1, TAGLN is less than that of copy number loss. The mutation positions of the 11 genes on the 23 pairs of chromosomes are shown in Figure 2D.

### 3.3 Analysis of consensus clustering and the immune microenvironmental landscape

**FIGURE 3:**
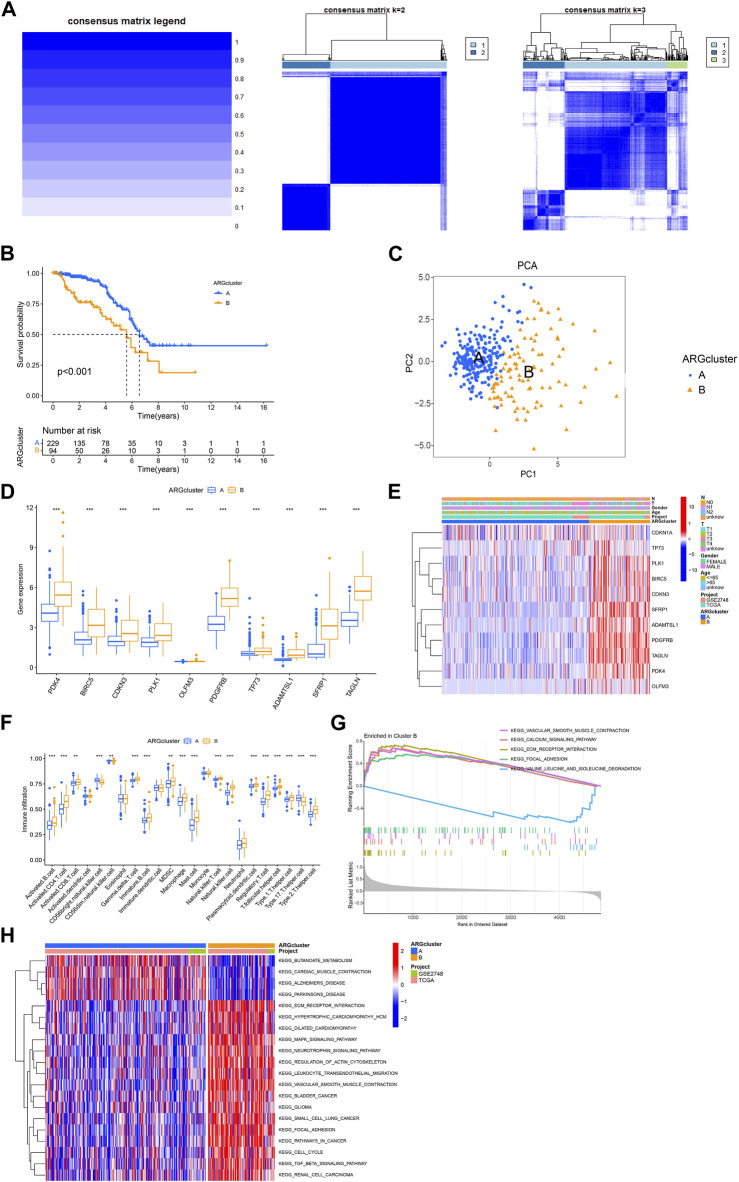
Analysis of consensus cluster and the immune microenvironmental landscape. **(A)** The consensus matrixes for the legend, k = 2 and k = 3 were obtained by applying consensus clustering. **(B)** Overall survival of two subtypes (*p* < 0.001). **(C)** Principal component analysis shows a significant distribution of patients in cluster A and cluster B based on the ARGs prognostic signature. **(D)** ARGs expression in two subtype clusters. **(E)** Heatmap of ARGs expression and corresponding clinicopathological features of two subtypes. **(F)** Immune infiltration patterns in two subtype clusters. **(G,H)** GSVA analysis focused on the differential enrichment of KEGG pathways between clusters B and A.

Next, we used the consensus cluster analysis to cluster KIRP patients into different subgroups based on 11 ARGs. The heatmap shows the best classification of KIRP patients with K = 2 (Figure 3A), with 229 samples in group A and 94 samples in group B, and the survival analysis of the two subgroups shows that cluster A outperforms cluster B in terms of survival time (Figure 3B), the PCA illustrates the significant separation between cluster A and cluster B based on 11 ARGs (Figure 3C). Differential analysis revealed these 11 genes were highly expressed in cluster B (Figure 3D), confirmed by a heatmap (Figure 3E). The difference analysis of immune cells between the two groups of samples was carried out. Results of the single-sample gene set enrichment analysis (ssGSEA) revealed 18 immune cells with significant differences between the two subtypes. Activated.B.cell, Activated. CD4.T.cell, Activated. CD8.T.cell, Gamma. delta.T.cell, Immature.B.cell, MDSC, Macrophage, Mast. cell, Natural. killer.T.cell, Natural. killer.cell, Plasmacytoid. dendritic.cell, Regulatory.T.cell, T. follicular.helper.cell, Type.1.T.helper.cell, Type.2.T.helper.cel were highly expressed in cluster B, whereas CD56bright.natural.killer.cell, CD56dim.natural.killer.cell, Type.17.T.helper.cell were highly expressed in cluster A (Figure 3F). Gene set variation analysis (GSVA) revealed differential enrichment of KEGG pathways between clusters B and A. KEGG_BUTANOATE_METABOLISM, KEGG_CARDIAC_MUSCLE_CONTRACTION, KEGG_ALZHEIMERS_DISEASE, and KEGG_PARKINSONS_DISEASE were highly enriched in cluster A, while the remaining pathways were highly enriched in cluster B (Figure 3H). Gene set enrichment analysis (GSEA) pathway analysis showed functional activity of KEGG_CALCIUM_SIGNALING_PATHWAY, KEGG_ECM_RECEPTOR_INTERACTION, KEGG_FOCAL_ADHESION, and KEGG_VASCULAR_SMOOTH_MUSCLE_CONTRACTION in cluster B, with KEGG_VALINE_LEUCINE_AND_ISOLEUCINE_DEGRADATION being functionally silent in cluster B (Figure 3G). Overall, these results suggest that ARGs may provide insights into the immune response and immune infiltration in KIRP.

### 3.4 Construction of a prognostic model

**FIGURE 4:**
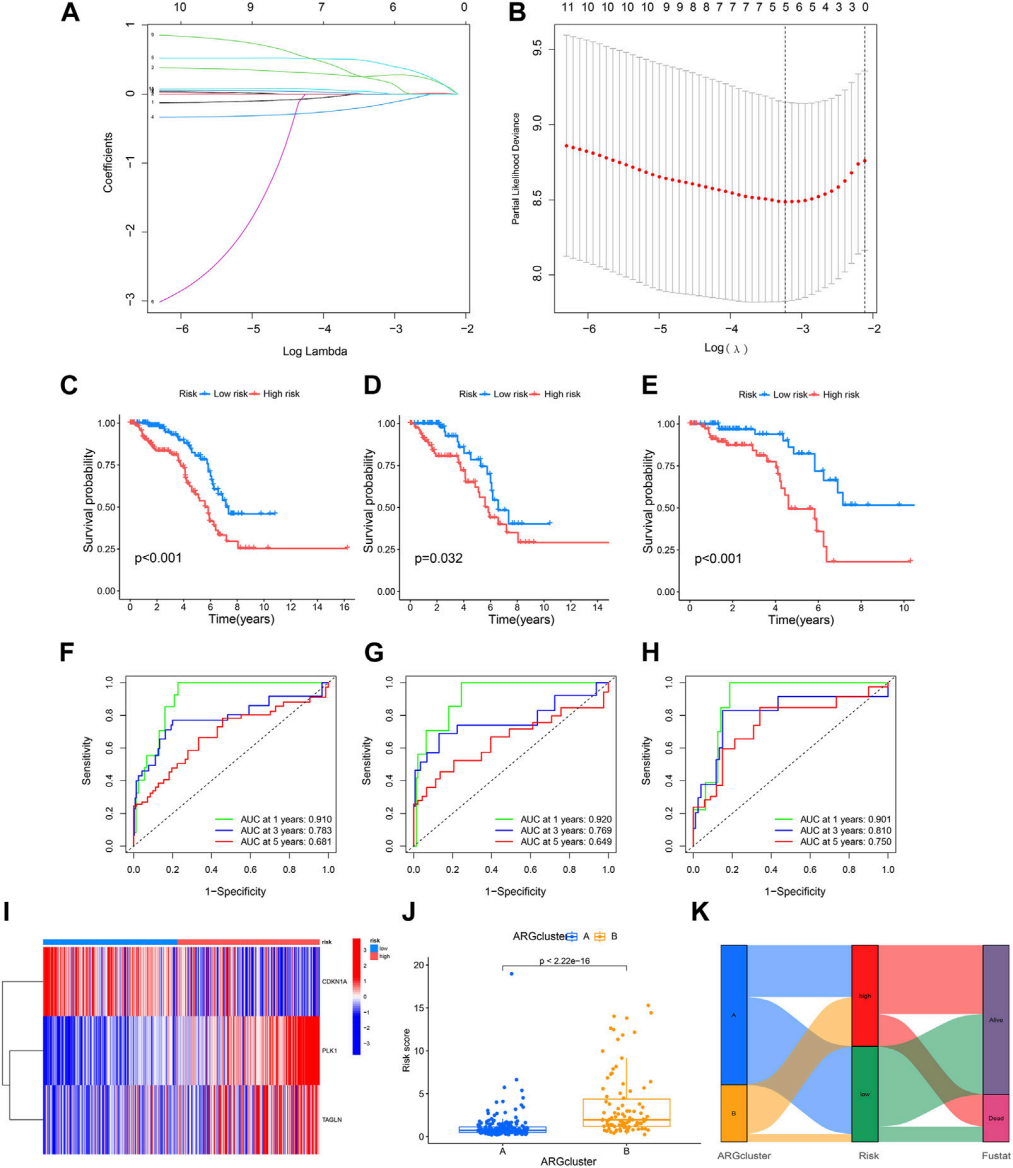
Construction of a prognostic model. **(A,B)** LASSO regression analysis shows the minimum lambda and optimal coefficients of the prognostic ARGs. **(C–E)** The K-M curves showed different prognosis in the different risk groups. **(C)** Train group, **(D)** Test group, **(E)** All group. **(F–H)** The time-dependent ROC curves for OS at 1-, 3-, and 5-years. **(F)** Train group, **(G)** Test group, **(H)** All group. **(I)** Heatmap diagram shows the expression of the 3 prognostic ARGs. **(J)** Risk score in two clusters established before. **(K)** Alluvial diagram of subtype and living status.

Five ARGs (CDKN3 CDKN1A PLK1 ADAMTSL1 TAGLN) associated with OS rates were identified by LASSO based on univariate Cox regression analysis. Based on multivariate Cox regression analysis, three ARGs (CDKN1A PLK1 TAGLN) that could independently predict the prognosis of KIRP were selected for constructing a risk model. According to the regression analysis results of these 3 prognostic genes, the risk score prognostic model was established as follows: (−0.373813566624579 * CDKN1A expression) + (0.884910890301322 * PLK1 expression) + (0.214132906470507 * TAGLN expression) (Figures 4A, B). The KIRP patients were randomly divided into two groups: a training group and a testing group, and according to the patient’s median risk score, patients were divided into high-risk and low-risk groups for survival analysis. The results showed that the survival rate of patients in the low-risk group was better than that in the high-risk group, which shows that the constructed model can accurately distinguish patients in high and low-risk groups (Figures 4C–E). The ROC curve indicated that the AUC values of 1-y, 3-y, and 5-year patients surviving in the training group are 0.901, 0.810, and 0.750 respectively, and the AUC values of 1-y, 3-y, and 5-year patients surviving in the testing group are 0.920, 0.769, 0.649, and the AUC values of 1-y, 3-y, and 5-year survival of patients in all groups were 0.910, 0.783, 0.681, indicating that the constructed model can accurately predict the patient’s survival period (Figures 4F–H). The risk heatmap showed that CDKN1A was a low-risk gene, while PLK1 and TAGLN were high-risk genes (Figure 4I). The difference analysis between clusters A and B shows that there is a significant difference in the patient’s risk score between the two groups (Figure 4J), and the corresponding correlation between the patients’ type and the survival status is shown in Figure 4K.

### 3.5 Constructing a nomogram

**FIGURE 5:**
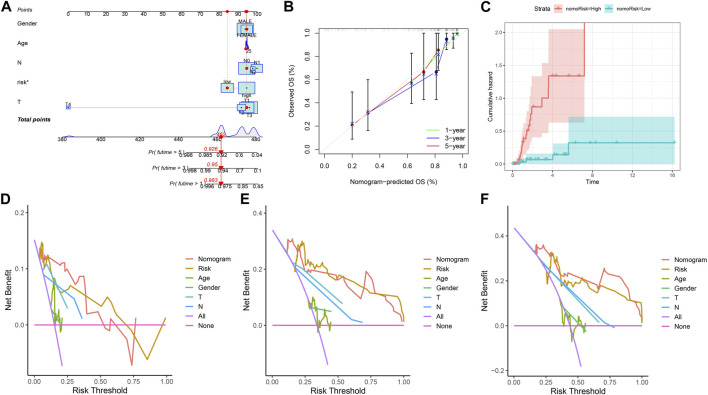
Constructing a nomogram. **(A)** Nomogram plot based on ARGscore and clinicopathological factors. **(B)** Calibration plot for the validation of the nomogram. **(C)** Cumulative hazard curve represented the probability of survival over time progression. **(D–F)** DCA curves of the nomogram for 1-, 3- and 5-year OS in KIRP patients.

In view of the role of the risk score in assessing patient survival, we used the risk score of the three genes combined with other clinicopathological features to construct a nomogram with the purpose of predicting the survival probability of KIRP. Each feature corresponds to a score, and a patient’s total risk score is 462 points. This indicates that the patient’s 1-year, 3-year, and 5-year survival rates are 0.983, 0.95, and 0.926, respectively. (Figure 5A). The predicted nomogram showed that the 1-year, 3-year, and 5-year OS curves were relatively well-revealed compared to the ideal model for the entire cohort (Figure 5B). Patients’ risk increases over time, and patients in the high-risk group had a greater risk than those in the low-risk group (Figure 5C). Decision curves demonstrated that the constructed nomogram was superior to other clinical traits in predicting patient survival (Figures 5D–F).

### 3.6 Immune cell infiltration and tumor microenvironment analysis

**FIGURE 6:**
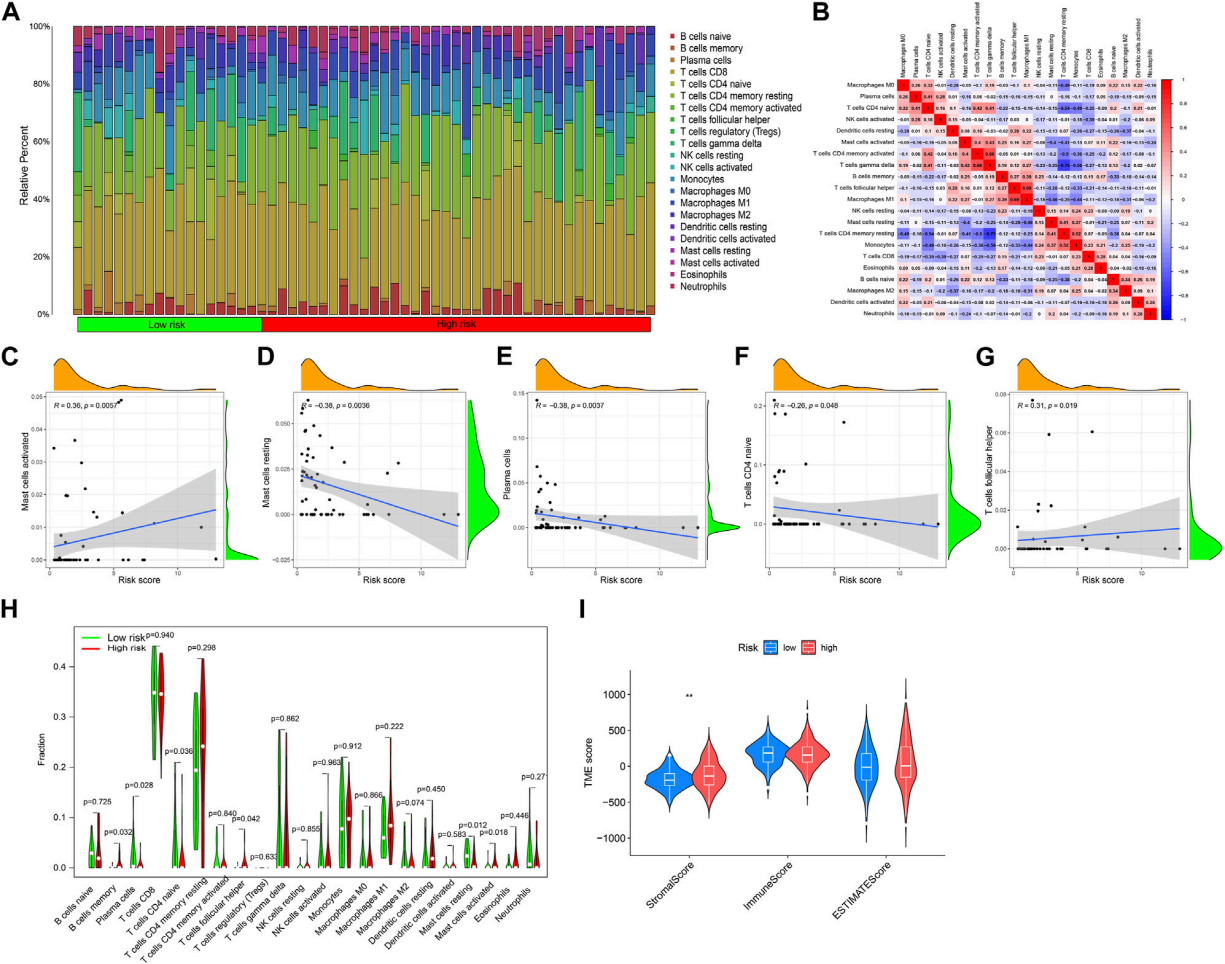
Immune cell infiltration and tumor microenvironment analysis. **(A)** The relative proportion of infiltrating immune cells with different risk scores. **(B)** Correlation between immune cells. **(C–G)** The correlation analysis between risk score and the proportion of immune cells in KIRP tissues. **(H)** Immune cell component between the high-risk group and low-risk group. **(I)** Estimate score of the expression profile in the high-risk group and low-risk group.

The immune response has an irreplaceable role in the anti-cancer process, so we evaluated the difference in the immune cells between the high and low-risk groups, and the content of immune cells was significantly different between the two groups (Figure 6A), and the correlation heatmap between immune cells is shown in Figure 6B. Figures 6C–G shows that B cells memory, Plasma cells, T cells CD4 naive, T cells focal helper, Mast cells resting, and Mast cells activated have significant differences between the two groups. The correlation analysis between immune cells and a patient’s risk score showed that T cells follicular helper and Mast cells activated were positively correlated with the risk score, while T cells CD4 naive, Plasma cells, and Mast cells resting were negatively correlated with the risk score (Figure 6H). The differential analysis of the tumor microenvironment revealed that there were significant differences in StromalScore between the high- and low-risk groups, and the tumor microenvironment (TME) score of the high-risk group was significantly higher than that of the low-risk group (Figure 6I).

### 3.7 Drug sensitivity analysis

**FIGURE 7:**
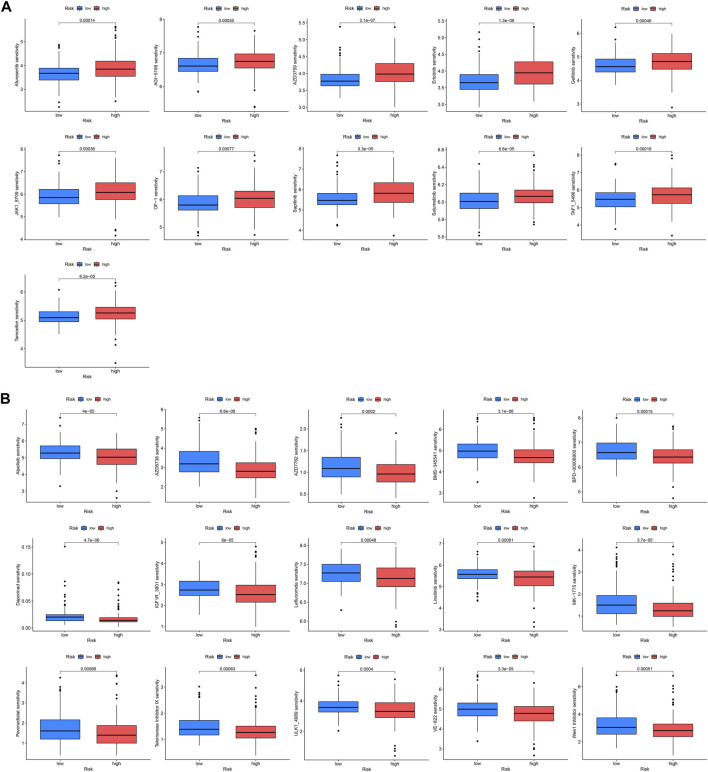
Drug sensitivity analysis. IC50 values were calculated for patients in the high- and low-risk groups to assess the sensitivity of chemotherapeutic agents. **(A)** Drugs that are highly sensitive in low-risk groups. **(B)** Drugs that are highly sensitive in high-risk groups.

Drug therapy is an indispensable part of tumor treatment, therefore, we conducted a drug sensitivity analysis related to prognostic genes for KIRP patients, and the results showed that 26 drugs had significant differences between high and low-risk groups. The IC50 values of these 11 drugs (AZD3759, Afuresertib, Gefitinib, Erlotinib, AGI−5198, BMS−345541, OF−1, JAK1_8709, Sapitinib, Selumetinib, Tamoxifen) were significantly increased in the high-risk group, indicating that these drugs sensitivity in the high-risk group was lower than that in the low-risk group (Figure 7A), and the IC50 values of these 15 drugs (Alpelisib, AZD6738, AZD7762, BPD−00008900, Daporinad, MK−1775, IGF1R_3801, Leflunomide, Linsitinib sensitivity, ULK1_4989, Telomerase Inhibitor IX, VE−822, Pevonedistat, TAF1_5496, Wee1 Inhibitor) were significantly lower in the high-risk group, indicating that these drugs are highly sensitive in the high-risk group (Figure 7B).

### 3.8 Screening of independent prognostic genes and their preliminary validation

**FIGURE 8:**
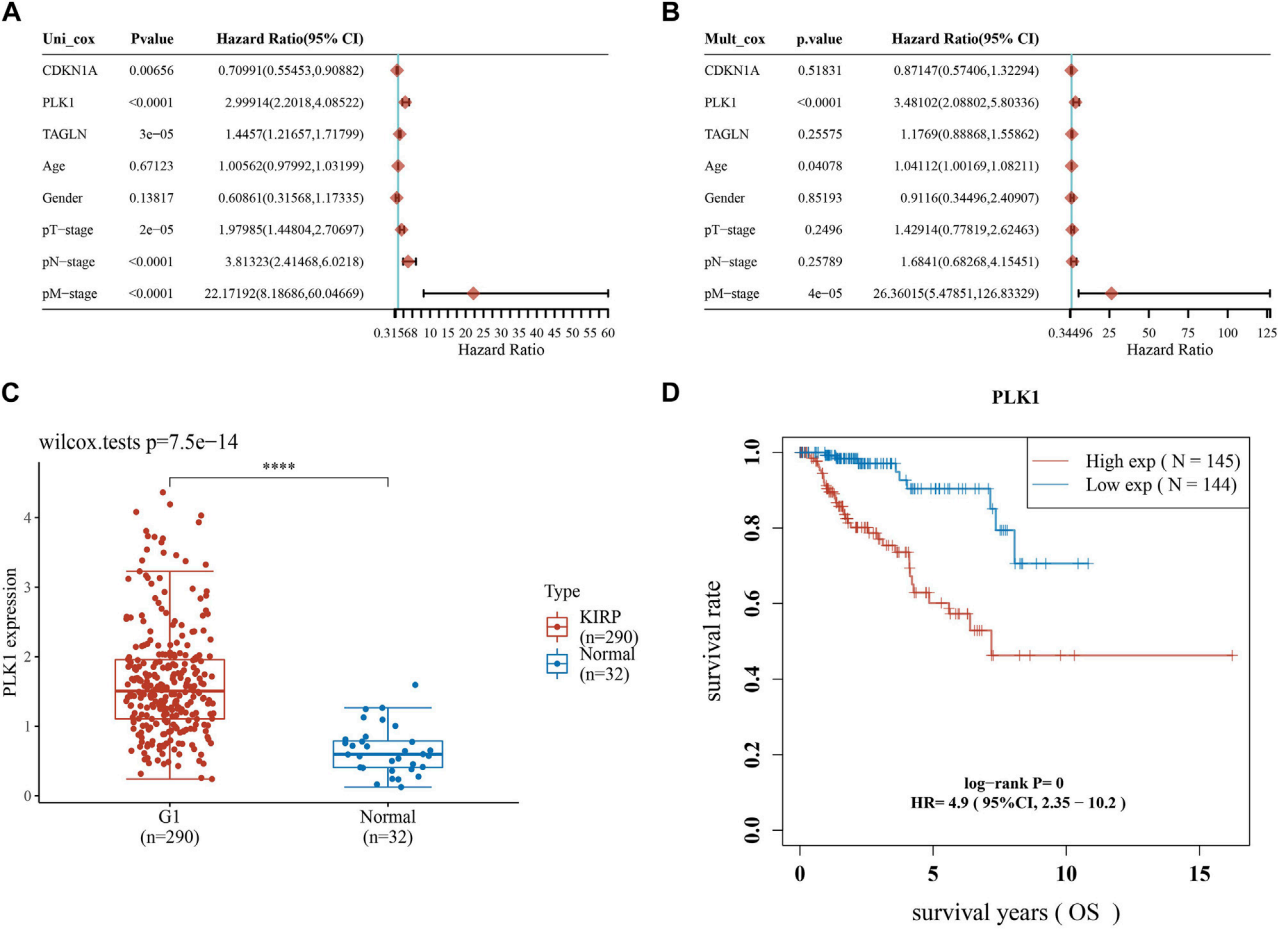
Screening for independent prognostic genes. **(A,B)** Hazard ratio and *p*-value of the constituents involved in univariate and multivariate Cox regression considering clinical parameters and three prognostic ARGs in KIRP. **(C)** Expression of PLK1 in KIRP and adjacent normal tissues. **(D)** Comparison of OS of PLK1 in KIRP high expression group and low expression group.

Afterwards, we performed univariate and multivariate Cox analysis of the *p*-values, risk coefficients HR and CI of the three prognostic gene expressions and clinical characteristics, and found that PLK1 and pM stages could be used as independent prognostic factors for KIRP (Figures 8A, B). The differential expression of PLK1 in adjacent normal tissues and KIRP tissues revealed that PLK1 was highly expressed in KIRP tissues (Figure 8C), and the high-expression group of PLK1 had a worse survival time than the low-expression group (Figure 8D).

### 3.9 Validation of PLK1 expression and prognostic function in KIRP

**FIGURE 9:**
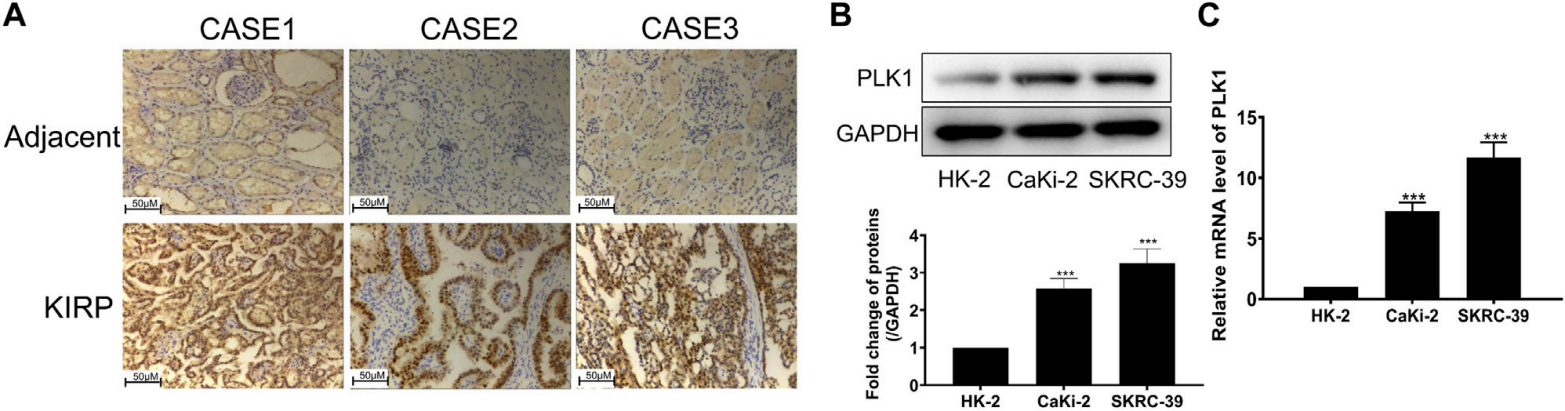
PLK1 is overexpressed in KIRP tissue and cells. **(A)** The expression of PLK1 in KIRP and adjacent tissues was determined by IHC. **(B)** The expression of PLK1 in two KIRP cells and one normal renal cell was determined by WB. **(C)** The expression of PLK1 in two KIRP cells and one normal renal cell was determined by PCR.

**FIGURE 10:**
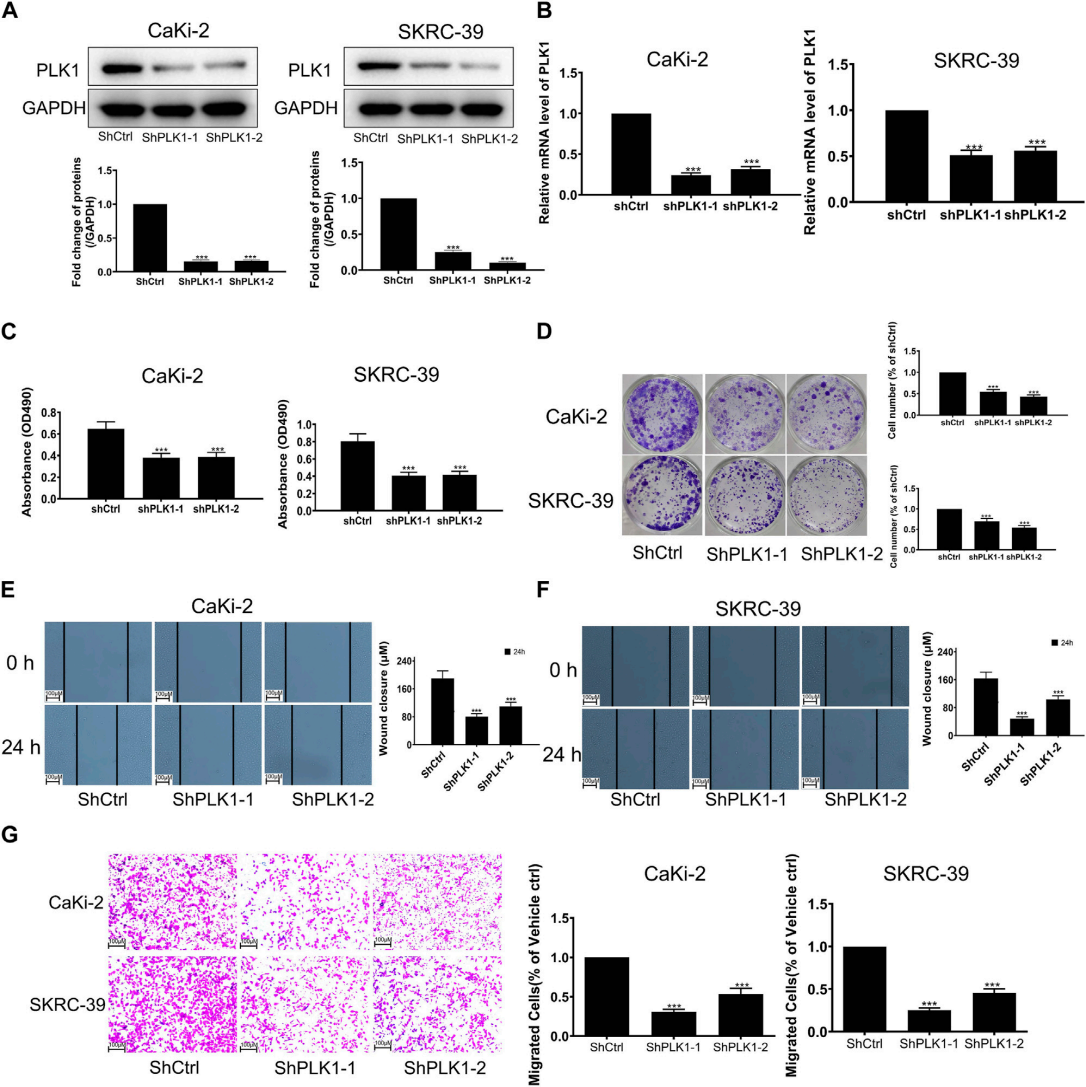
Validation of PLK1 prognostic function in KIRP. **(A)** The expression of PLK1 after silencing PLK1 was measured by WB. **(B)** The expression of PLK1 after silencing PLK1 was measured by PCR. **(C)** MTT was used to evaluate cell proliferation after silencing PLK1. **(D)** Clonogenic assay was used to evaluate colony suppression after silencing PLK1. **(E–G)** Scratch and transwell assay was used to evaluate migration suppression after silencing PLK1.

To investigate the expression of PLK1 in KIRP, we stained PLK1 on adjacent normal tissues and KIRP tissues. IHC results showed that PLK1 was significantly higher in KIRP compared with adjacent normal tissues (Figure 9A). Next, we evaluated PLK1 expression in two KIRP cell lines and one normal renal cell line by WB and qRT-PCR. As shown in Figures 9B, C, we found that the protein and mRNA levels of PLK1 in KIRP were significantly higher than those in normal cells. Therefore, we further explored the function of PLK1 in KIRP. After the successful silencing of PLK1 was verified by WB and PCR (Figures 10A, B), the effects of PLK1 on KIRP were respectively detected by MTT, clonogenic, scratch, and transwell assays, and the results showed that silencing PLK1 significantly inhibited cell proliferation, clonogenesis and migration (Figures 10C–G). All of these results suggest that PLK1 plays an important role in the proliferation and migration of KIRP.

## 4 Discussion

Given the extremely poor prognosis once KIRP is diagnosed, the 5-year survival rate is significantly low. Therefore, early diagnosis and risk assessment are very important to improve the survival time of KIRP patients. In our study, 3 ARGs were identified to be associated with KIRP’s OS, and they were used to construct a new risk model to assess the prognosis of KIRP. The role of prognosis-related genes in KIRP was further evaluated through immune cell infiltration and drug sensitivity analyses. Basic experiments further verified the expression and function of the independent prognostic gene PLK1.

First, we obtained 11 genes (PDK4, BIRC5, CDKN3, CDKN1A, PLK1, OLFM3, PDGFRB, TP73, ADAMTSL1, SFRP1, TAGLN) associated with KIRP prognosis from 99 DEGs by univariate regression analysis. Pyruvate dehydrogenase kinase 4 (PDK4) is one of the important biomarkers involved in energy metabolism, which is related to the energy metabolism of proximal tubule cells and involved in the occurrence and development of chronic kidney disease (CKD) (Zhou et al., 2022). The occurrence of CKD can cause the decline of renal function, which has a higher prognosis correlation with KIRP, and the aggravation of renal injury can accelerate the occurrence and development of KIRP (Woldu et al., 2014). Baculovirus IAP repeats 5 (BIRC5) is a member of the inhibitor of apoptosis (IAP) family and plays an important role in the occurrence and development of tumors. BIRC5 is highly expressed in most cancers and has a strong oncogenic effect in promoting cancer cell proliferation and cancer development, which significantly shortens the survival time of patients (Ye et al., 2022). A recent study demonstrated that BIRC5 expression is upregulated in kidney renal clear cell carcinoma (KIRC), and promotes KIRC proliferation and tumorigenicity (Zhang et al., 2019). Small nucleolar RNA host gene 6. (SNHG6) has been shown to be associated with poor prognosis of various human cancers (Shen et al., 2020), which may regulate BIRC5 expression to promote KIRP progression (Liu et al., 2023). BIRC5 was also proved to be the prognosis-related gene of KIRP in our study, further indicating that the expression of BIRC5 is crucial to the occurrence and development of KIRP. Cyclin-dependent kinase inhibitor 3 (CDKN3) has also been proven to be related to the occurrence and treatment of various cancers, such as bladder carcinoma, colorectal carcinoma, cervical carcinoma, *etc.* (Berumen et al., 2014; Li et al., 2020; Li et al., 2022), However, its role in KIRP has not been elucidated so far, and it is the first time in our paper that CDKN3 is associated with the prognosis of KIRP.

Through GSVA and GSEA analysis to explore the activity of KEGG pathways in clusters A and B, it was found that KEGG_ECM_RECEPTOR_INTERACTION was active in cluster B, while KEGG_VALINE_LEUCINE_AND_ISOLEUCINE_DEGRADATION was silent in cluster B. The expression of ITGA6 and CD44 in the ECM-receptor-interaction pathway plays an important role in the proliferation and invasion of renal cell carcinoma cells. Treatment offers a new research direction (Zhang et al., 2016). Increased concentrations of leucine, isoleucine, and valine were found in the urine of patients with non-small cell lung carcinoma treated with cisplatin infusion, suggesting that valine leucine and isoleucine degradation may be affected by cisplatin influence (Doskocz et al., 2015). Therefore, the analysis of GSVA and GSEA can provide a more scientific basis for the immunotherapy and chemotherapy of KIRP.

Subsequently, we identified three genes (CDKN1A, PLK1, TAGLN) that can independently predict the prognosis of KIRP. These genes were selected using the LASSO algorithm via univariate and multivariate Cox regression analysis, and were then used to construct a risk model, in general, the constructed risk model can accurately predict the prognosis of KIRP patients.

The cyclin-dependent kinase inhibitor p21 (CDKN1A) plays an important role in the DNA damage response by inducing cell cycle arrest, directly inhibiting DNA replication, and regulating fundamental processes such as apoptosis and transcription (Cazzalini et al., 2010). The cell cycle is a complex process that is regulated by a large number of genes at the transcriptional, post-transcriptional, and post-translational levels, and abnormalities at any stage of the cell cycle may lead to diseases including cancer. Therefore, genes play a crucial role in the proliferation process role. CDKN1A is the target of miRNA, and its expression can affect the binding site of transcription factors in the promoter region, thereby affecting the proliferation of DNA, causing cell generation obstacles, and possibly inducing cancer (Bhattacharyya et al., 2016). Until now, CDKN1A has been found to play a role in numerous cancers. CDKN1A can promote ovarian cancer resistance to cisplatin, resulting in cisplatin treatment failure and aggravating the patient’s condition (Wang and Liu, 2021). The study found that the mRNA and protein expression levels of CDKN1A/p21 were significantly upregulated in breast carcinoma tissues compared with adjacent non-neoplastic breast tissues, which indicated that high expression of CDKN1A/p21 was closely related to adverse pathological parameters and poor prognosis of breast carcinoma, and CDKN1A/p21 can be listed as a possible candidate for breast carcinoma biomarker (Zaremba-Czogalla et al., 2018). Many studies have found that microRNA (miR)-93 can play an oncogenic role in various cancers by inhibiting the expression of CDKN1A. Compared with normal tissues, the expression levels of miR-93 and CDKN1A in cervical carcinoma tissues were significantly increased and decreased, indicating that the upregulation of miR-93 and the downregulation of CDKN1A may be related to the occurrence and development of cervical carcinoma and the prognosis of patients, suggesting that CDKN1A could potentially serve as a therapeutic target for this carcinoma (Zhang et al., 2018). CDKN1A acts as a cell cycle regulator involved in genome stability, and low expression of CDKN1A contributes to poor prognosis in chromophobe renal cell carcinoma (Ohashi et al., 2020). CDKN1A has been shown to be a member of ferroptosis-associated signature genes to predict graft loss after renal allograft transplantation (Fan et al., 2021). Moreover, the prognosis of KIRP patients can also be accurately predicted by the risk model constructed by using CDKN1A as a ferroptosis-related gene signature (Da et al., 2022). A study in a mouse model identified CDKN1A as a target of phagocytosis-mediated immunotherapy of acute leukemia cells, CDKN1A promotes phagocytosis of leukemia cells and subsequently promotes pro-inflammatory reprogramming of phagocytic macrophages via interferon Gamma extends to surrounding macrophages, presenting the therapeutic opportunity for cancer therapy (Allouch et al., 2022). However, in our study, CDKN1A was used for the first time as an anoikis-related gene to construct a risk model for KIRP. This model helps to evaluate the prognosis and immune response of KIRP patients, thereby further expanding the understanding of CDKN1A’s role in KIRP.

Polo-like kinase 1 (PLK1) is an evolutionarily conserved Ser/Thr kinase, which plays an important role in the regulation of the cell cycle and is mainly expressed in the G2/S and M phases of the cell cycle (Petronczki et al., 2007). Genetic ablation or inhibition of PLK1 results in abnormal chromosome segregation and, consequently, mitotic arrest, often accompanied by cell death (von Schubert et al., 2015). PLK1 is often overexpressed in a variety of tumors and is associated with poor clinical outcomes. In our study, we found that the expression of PLK1 was increased in KIRP, and the prognosis of high expression of PLK1 was worse than that of low expression. In addition, PLK1 overexpression is associated with chemotherapy resistance, and inhibition of PLK1 can enhance the sensitivity of cancer cells to chemotherapy and radiotherapy (Strebhardt, 2010; Gutteridge et al., 2016). In non-small cell lung carcinoma (NSCLC), inhibition of PLK1 selectively kills cancer cells and upregulates PD-L1 expression in surviving cancer cells, providing a feed-forward target for antigen-releasing agents and checkpoint inhibitors (ARACs) Delivery provides the opportunity to effectively reach immunotherapy in NSCLC (Reda et al., 2022). Similarly, a study suggests that Plk1 may be a biomarker of gemcitabine response in pancreatic ductal adenocarcinoma (PDAC), inhibition or depletion of Plk1 leads to upregulation of PD-L1 via activation of the NFκB pathway. Plk1 can thus co-inhibit PDAC progression and suppress NFκB activity. Consequently, targeting Plk1 could enhance the efficacy of PDAC immunotherapy (Zhang et al., 2022). The PLK1 gene is a potential anti-breast cancer drug target and a prognostic marker, and its overexpression is closely related to the low survival rate of breast carcinoma patients (Fang et al., 2022). As a necroptosis-related gene, PLK1 has also been studied in KIRC, and it was found that PLK1 can be used as an independent prognostic marker for KIRC patients with excellent stability and accuracy (Xin et al., 2022). Although some studies have also found that PLK1 can be used as a new important prognostic factor involved in the pathogenesis of KIRP (He et al., 2020), but these are not very comprehensive, and further research is needed. In our study, PLK1, as one of the independent prognostic factors of KIRP, played an indispensable role in KIRP. We conducted qRT-PCR and WB experiments on PLK1 and found that the protein and mRNA levels of PLK1 in KIRP were significantly higher than those in normal cells, which was consistent with the differential expression of KIRP, further proving the accuracy of our results. We also further explored the function of PLK1 in KIRP and found that silencing PLK1 significantly inhibited cell proliferation, clonogenicity, and migration, suggesting that PLK1 plays an important role in the regulation of the cell cycle and the proliferation and migration of KIRP.

Transgelin (TAGLN, also known as SM22), an actin-associated protein, is found to have a significant role in the development of various cancers due to its dysregulation. It is generally considered a tumor suppressor (Wen et al., 2021) and has an important role. Studies have identified TAGLN as a potential molecular target for colorectal cancer progression, as the interaction between TAGLN and HMGA2 is involved in TGF-*β*-induced cell migration and promotion of colon carcinoma cells (Zhou et al., 2020). TAGLN has also been shown to be a potential biomarker for diagnostic and therapeutic targets, significantly impacting the survival of bladder carcinoma patients (Liu et al., 2019). The evaluation of the response to immunotherapy and chemotherapy in patients with bladder carcinoma by the prognostic model constructed by TAGLN showed that the high-risk group showed significantly higher sensitivity to anti-PD-1 therapy (Liu et al., 2021). There is no relevant evidence for TAGLN as a prognostic biomarker in KIRP. In our study, TAGLN, as a gene that can independently predict the prognosis of KIRP, participated in the construction of the risk model. Overall, it is suggested that the constructed model can accurately predict the prognosis of KIRP, further reinforcing its role in KIRP. It also provides a new potential target for the future treatment and research of KIRP.

Our drug sensitivity analysis revealed 15 drugs sensitive, and 11 drugs insensitive, to the high-risk group. Afuresertib is an Akt pathway inhibitor that exerts cytotoxic and antiproliferative activities in human cancer cells (Uko et al., 2020). And in the study in rats, it was also found that Afuresertib can inhibit the expression of PI3K and Akt-related proteins in rat tumor tissues to exert its anti-tumor effect (Min et al., 2022). These show that Afuresertib has anti-tumor effects in both humans and animals. Erlotinib (OSI-774) inhibits the epidermal growth factor receptor (EGFR), which blocks tumor cell division, produces cell cycle arrest, and initiates programmed cell death in EGFR-overexpressing human tumor cells, and has therapeutic potential for lung cancer strong effect (Brower and Robins, 2016; Tagliamento et al., 2018). However, the therapeutic efficacy of these drugs for KIRP remains largely unexplored. In our study, the sensitivity of these drugs in KIRP was analyzed, which provides more potential options for drug treatment of KIRP.

Although the characteristic model of ARGs that we constructed has strong performance in predicting the prognosis of KIRP patients, there are still many limitations in our study. The KIRP samples used in our research are mainly sourced from public databases, which may lack some important information. Although we also collected some clinical tissue samples for basic experimental verification, our research results still need more *in vivo* and *in vitro* experiments for further verification. In addition, our prognostic model was constructed by screening only a few characteristic risk genes, while other valuable genes may be ignored. More research is needed to further verify our results.

## 5 Conclusion

We identified 99 DEGs associated with KIRP survival, from which we selected 3 genes to construct a prognostic model that effectively predicts the prognosis of KIRP patients. Furthermore, the relationship between different subtypes and immune microenvironments and different functions was initially demonstrated by immune infiltration landscape analysis and functional enrichment analysis. Drug sensitivity analysis screened 15 highly sensitive drugs in the high-risk group and 11 highly sensitive drugs in the low-risk group. PLK1 is an independent prognostic factor for KIRP, and its mRNA and protein expression levels are consistent with gene differential expression levels, both of which are highly expressed in KIRP. The results of functional verification in KIRP found that silencing PLK1 significantly inhibited cell proliferation, clonogenesis, and migration, indicating PLK1’s importance in KIRP. In conclusion, our findings can provide a new idea for future research and provide clinicians with new targets for the treatment of KIRP patients.

## Data Availability

The original contributions presented in the study are included in the article/Supplementary Material, further inquiries can be directed to the corresponding authors.
