# Supplementary material for: Role of anoikis-related gene PLK1 in kidney renal papillary cell carcinoma: a bioinformatics analysis and preliminary verification on promoting proliferation and migration
Source: Front Pharmacol. 2023 Jun 29;14:1211675. doi: 10.3389/fphar.2023.1211675 (PMC10339314; doi:10.3389/fphar.2023.1211675)
Supplement: Supplementary file 1 [file DataSheet1.docx]

Supplementary Material

**Supplementary Figure S1.** The protein-protein interaction (PPI) of 99 DEGs.

**Supplementary Table S1.** The clinical characteristics of KIRP patients in the TCGA cohort.

| Clinical characters | Number |
| --- | --- |
| Gender  Male  Female | 214  76 |
| Age |  |
| Mean (SD) | 61.4 (12) |
| Median [MIN, MAX] | 61 [28, 88] |
| TNM stage  I  II  III  IV | 179  25  52  16 |
| pT_stage  T1  T2  T3  T4  TX | 199  37  48  2  4 |
| pN_stage  N0  N1  N2  NX | 145  24  3  118 |
| pM_stage  M0  M1  MX | 205  10  75 |

**Supplementary Table S2.** 640 genes were downloaded from the Genecards database and Harmonizome database.

| Gene |
| --- |
| BMF |
| DAPK2 |
| E2F1 |
| STK11 |
| TFDP1 |
| ABHD4 |
| AFAP1L1 |
| AKT1 |
| AKT2 |
| ANGPTL4 |
| BAX |
| BCAR1 |
| BCL2 |
| BCL2L11 |
| BIRC3 |
| BMF |
| BRAF |
| BRCA2 |
| BSG |
| CALR |
| CASP2 |
| CASP3 |
| CASP8 |
| CAV1 |
| CCAR2 |
| CD63 |
| CDCP1 |
| CDH1 |
| CDH2 |
| CDKN2A |
| CEACAM6 |
| CEBPB |
| CHUK |
| CLDN1 |
| CLU |
| CMA1 |
| COPS5 |
| CSNK2A1 |
| CSPG4 |
| CTNND1 |
| CTTN |
| CXCL12 |
| DAP3 |
| DAPK1 |
| DLG1 |
| EDA2R |
| EEF1A1 |
| EEF2K |
| EGFR |
| EIF2AK3 |
| ERBB4 |
| FER |
| FGF2 |
| FN1 |
| HGF |
| HK2 |
| HMCN1 |
| HMGA1 |
| HOXA10 |
| HTRA1 |
| IGF1R |
| IKZF3 |
| ITGA2 |
| ITGA3 |
| ITGA4 |
| ITGA5 |
| ITGA6 |
| ITGA8 |
| ITGAV |
| ITGB1 |
| KDR |
| KL |
| KRAS |
| LGALS1 |
| LRP1 |
| LTB4R2 |
| MAPK1 |
| MAPK3 |
| MAVS |
| MCL1 |
| MDM2 |
| MET |
| MGAT5 |
| MIR200C |
| MMP11 |
| MMP13 |
| MMP2 |
| MTA1 |
| MTOR |
| MYBBP1A |
| NRP1 |
| NTF3 |
| NTRK2 |
| OLFM3 |
| PAK1 |
| PAK4 |
| PECAM1 |
| PIK3CA |
| PIK3CG |
| PLK1 |
| PRKCA |
| PRKD1 |
| PTEN |
| PTHLH |
| PTK2 |
| PTK2B |
| PTK6 |
| PTPN11 |
| PTRH2 |
| RAD9A |
| RHOA |
| RHOC |
| RIPK1 |
| ROCK1 |
| S100A4 |
| SCRIB |
| SH3GLB1 |
| SIK1 |
| SIRPA |
| SIRT3 |
| SKP2 |
| SLCO1B3 |
| SMAD4 |
| SNAI2 |
| SRC |
| STAT3 |
| STK11 |
| TAGLN |
| TGFB1 |
| THBS1 |
| TIMP1 |
| TP53 |
| TPM1 |
| UCHL1 |
| USP9X |
| WISP3 |
| WNT2 |
| XIAP |
| YWHAZ |
| BRMS1 |
| PTK2 |
| NTRK2 |
| BCL2L11 |
| SRC |
| CEACAM6 |
| CAV1 |
| AKT1 |
| ITGB1 |
| CEACAM5 |
| EGFR |
| BCL2 |
| CASP8 |
| SIK1 |
| PTRH2 |
| STAT3 |
| TLE1 |
| DAPK2 |
| CTNNB1 |
| ZNF304 |
| MAPK1 |
| BMF |
| ITGA5 |
| TP53 |
| MCL1 |
| BCL2L1 |
| CASP3 |
| CDH1 |
| BAD |
| PIK3CA |
| PAK1 |
| ITGAV |
| FN1 |
| MAPK3 |
| PTGS2 |
| BAX |
| BCAR1 |
| PTEN |
| ERBB2 |
| ANGPTL4 |
| PDK4 |
| CYCS |
| BRAF |
| YAP1 |
| ANKRD13C |
| ITGA2 |
| ANXA5 |
| BIRC5 |
| MTOR |
| TIMP1 |
| BDNF |
| CSPG4 |
| BSG |
| AKT2 |
| STK11 |
| IGF1 |
| IGF1R |
| ITGA6 |
| ILK |
| CFLAR |
| RHOA |
| HIF1A |
| DAP3 |
| MYBBP1A |
| TLE5 |
| ITGA3 |
| PTK2B |
| CCND1 |
| CTTN |
| CALR |
| ATF4 |
| CDCP1 |
| PLAUR |
| SKP2 |
| CHEK2 |
| HGF |
| E2F1 |
| EGF |
| PIK3CG |
| ITGB4 |
| DAPK1 |
| MAPK8 |
| PIK3R1 |
| PIK3R3 |
| MAP2K1 |
| CXCL12 |
| LGALS3 |
| FBXW7-AS1 |
| BAK1 |
| ABHD4 |
| CD44 |
| ITGA4 |
| FADD |
| PHLDA2 |
| TGFB1 |
| HMCN1 |
| MMP2 |
| CEBPB |
| CEMIP |
| CDKN3 |
| CBL |
| CASP9 |
| SFN |
| MTDH |
| PRKCA |
| TNFRSF10B |
| CXCL8 |
| MIR200C |
| AR |
| CDKN2A |
| CPT1A |
| PIK3CB |
| CLDN1 |
| MIR204 |
| MIR26A1 |
| CDKN1A |
| CDKN1B |
| KLF12 |
| NTRK1 |
| PLAU |
| MYC |
| SMAD4 |
| PLK1 |
| MUC1 |
| LGALS1 |
| PYCARD |
| SESN2 |
| ITGB3 |
| KRAS |
| THBS1 |
| BID |
| HRAS |
| CDK11B |
| CDK11A |
| XIAP |
| PPARG |
| IL6 |
| MIR145 |
| CCR7 |
| MSLN |
| RAC1 |
| GRHL2 |
| BIRC3 |
| NOTCH1 |
| RHOG |
| CCAR2 |
| NQO1 |
| MMP13 |
| FAS |
| MTA1 |
| MYO5A |
| EDA2R |
| CCN6 |
| MMP9 |
| ABL1 |
| MAPK11 |
| SOD2 |
| PTHLH |
| PDGFB |
| GLI2 |
| EZH2 |
| RIPK1 |
| CXCR4 |
| HMGA1 |
| SIK2 |
| TNFSF10 |
| ANGPTL2 |
| S100A4 |
| NTF3 |
| ETV4 |
| MIR21 |
| MIR124-1 |
| HTRA1 |
| LATS1 |
| CEACAM3 |
| EIF2AK3 |
| LAMC2 |
| LAMA3 |
| LAMB3 |
| CDH2 |
| CSNK2A1 |
| EDIL3 |
| ZEB2 |
| TLN1 |
| EPHA2 |
| SIRT3 |
| OLFM3 |
| CLU |
| SPINK1 |
| CPEB2 |
| NAT1 |
| TSG101 |
| MIR200A |
| MIR6744 |
| SERPINA1 |
| AKT3 |
| RELA |
| TNFRSF1A |
| FASLG |
| AFP |
| ITGA8 |
| NOX4 |
| PBK |
| SATB1 |
| CD63 |
| EEF1A1 |
| LTB4R2 |
| MAVS |
| HRC |
| CCN2 |
| RHOB |
| PPP1R13B |
| PLG |
| MET |
| RAF1 |
| PARP1 |
| PRKCQ |
| BRCA2 |
| RB1 |
| SP1 |
| HAVCR2 |
| DOCK1 |
| VTN |
| INHBB |
| PDCD4 |
| PRPF4B |
| RANBP9 |
| SESN1 |
| SESN3 |
| CD24 |
| ZBTB7A |
| MIR141 |
| ELANE |
| KDR |
| MDM2 |
| NFE2L2 |
| ZEB1 |
| KL |
| PRKCI |
| CRYAB |
| EPHB6 |
| FGF2 |
| HK2 |
| LTF |
| IQGAP1 |
| MGAT5 |
| SDCBP |
| ABHD2 |
| SPIB |
| TRIM31 |
| MIR1827 |
| PDGFRB |
| PLAT |
| TLR3 |
| NRAS |
| ROCK1 |
| PAK4 |
| VEGFA |
| CASP10 |
| PIN1 |
| IL1RAP |
| UBE2C |
| YWHAZ |
| TWIST1 |
| BMP6 |
| BNIP3L |
| ELK1 |
| KDM3A |
| PRDX4 |
| BNIP3 |
| LMO3 |
| ZNF32 |
| MIR200B |
| MIR525 |
| MIR363 |
| TUBB3 |
| HSP90B1 |
| SLC2A1 |
| HMOX1 |
| PTPN11 |
| PRKACA |
| PAK3 |
| CD36 |
| PIK3R2 |
| PPP2CA |
| CASP6 |
| CDH3 |
| EEF2K |
| LRP1 |
| PAK2 |
| PTK6 |
| LPAR1 |
| TCF7L2 |
| CEACAM1 |
| GDF2 |
| GLO1 |
| IL17A |
| RBL2 |
| SIRPA |
| TRAF2 |
| ADCY10 |
| VPS37A |
| TNFRSF12A |
| APOBEC3G |
| BAG1 |
| COL13A1 |
| MNX1 |
| RAD9A |
| IFI27 |
| MEGF11 |
| ITPRIP |
| BCL2L15 |
| SNAI2 |
| PTPN1 |
| NOTCH3 |
| GLUD1 |
| SIRT1 |
| FASN |
| MYH9 |
| RPS6KB1 |
| TPM1 |
| PPP2R1A |
| COL4A2 |
| CTNND1 |
| CD151 |
| MMP11 |
| ARHGEF7 |
| PPP2R2A |
| SEMA7A |
| PPP2R5A |
| BST2 |
| CCN1 |
| PPP2R2D |
| CCDC178 |
| MIR10A |
| MIR30B |
| MIR30C1 |
| SHC1 |
| BUB1 |
| CDC25C |
| CDK1 |
| ITGB5 |
| SETD2 |
| BUB3 |
| FER |
| TP73 |
| SLCO1B3 |
| TDGF1 |
| DLG1 |
| EDAR |
| MAD2L1 |
| BCL2L2 |
| PDCD6IP |
| SH3GLB1 |
| SCRIB |
| DYNLL2 |
| TSC2 |
| BAG4 |
| MAP3K7 |
| F10 |
| F3 |
| ADAMTSL1 |
| SERPINB1 |
| MIR181A1 |
| MAP3K1 |
| CTBP1 |
| CEACAM4 |
| PXN |
| MALAT1 |
| GSTP1 |
| PRDX1 |
| IKBKG |
| TFDP1 |
| CRYBA1 |
| SERPINE1 |
| FOXO3 |
| ACTG1 |
| ARHGDIA |
| EZR |
| SLC39A6 |
| BIN1 |
| TIAM1 |
| PDPK1 |
| SMAD7 |
| NTRK3 |
| RHOC |
| CASP2 |
| TNC |
| IRF6 |
| HOTAIR |
| GNE |
| XAF1 |
| SFRP1 |
| MAP2K2 |
| CSK |
| PIK3C2B |
| TAGLN |
| ENDOG |
| FOXC2 |
| RACK1 |
| ARHGDIB |
| FBLIM1 |
| CCDC80 |
| PRKD1 |
| LDHA |
| ANXA2 |
| SPP1 |
| SMARCE1 |
| QSOX1 |
| RBFOX2 |
| RPS6KA3 |
| CDC42 |
| MAOA |
| PIP5K1C |
| ATF2 |
| JUP |
| NDRG1 |
| NKX2-1 |
| OCLN |
| CRABP2 |
| ID2 |
| CEACAM8 |
| PITPNC1 |
| AFAP1L1 |
| INSR |
| HSPB1 |
| NGF |
| PCNA |
| GSK3B |
| TP63 |
| KRT14 |
| SPHK1 |
| CTNNA1 |
| EHMT2 |
| OGT |
| RAC3 |
| SIRT6 |
| ACP1 |
| FOXA1 |
| STK38 |
| RHOQ |
| ONECUT1 |
| S100A7 |
| SRSF3 |
| MUC4 |
| GKN1 |
| MIR107 |
| MIR630 |
| DNMT1 |
| LCK |
| MERTK |
| UCHL1 |
| CDK2 |
| MMP3 |
| ACTB |
| BRCA1 |
| SLC2A2 |
| NOS2 |
| USP9X |
| ROR1 |
| FYN |
| HSPA1A |
| HTRA2 |
| SNAI1 |
| C5AR1 |
| LATS2 |
| PRDM1 |
| SKI |
| TPP2 |
| XRCC5 |
| CLDN18 |
| SPTA1 |
| THY1 |
| TJP1 |
| CDX2 |
| CENPF |
| DOK2 |
| S100A11 |
| SERPINB5 |
| CLIC4 |
| IKZF3 |
| SNCG |
| USP11 |
| ELAVL1 |
| HOXA10 |
| LGALS8 |
| SRPX2 |
| SLPI |
| HTRA3 |
| EFHD2 |
| IRX1 |
| CXCL14 |
| KIF18A |
| ZG16B |
| SBSN |
| MIR223 |
| MIR503 |
| MIR99A |
| MIR451A |
| MIR7-1 |
| SNORA80E |
